# Is it exhausting to be a healthcare worker these days?

**DOI:** 10.1192/j.eurpsy.2022.771

**Published:** 2022-09-01

**Authors:** M. Solis, M. Valverde Barea, E. Perdiguero Sempere

**Affiliations:** Universitary Hospital of Jaén, Psychiatric, Jaén, Spain

**Keywords:** Stress, personal demotivation, healthcare workers, mental health

## Abstract

**Introduction:**

People spend a large part of our lives in the workplace. Stress at work, desmotivation and mental exhaustation are consequences derived from stressful situations that can be generated at work.

**Objectives:**

Detection in hospitals of those workers susceptible to exhaustion, work stress or personal demotivation to avoid a problem in the worker’s mental health, allowing early intervention and health strategies.

**Methods:**

A cross-sectional study was carried out that included 84 healthcare workers from Spain in October 2021, through an anonymous, voluntary and multiple response type online survey which included questions about sociodemographic aspects and the Maslach burnout inventory

**Results:**

62% were doctors and 29% were nurses, 3 workers were nursing assistants, 2 orderlies, 1 psychologist and 1 physiotherapist. 13% of workers report having received / thought about requesting care from a mental health team (psychologist / psychiatrist) in the last year. 8% admit to having had suicidal ideas in the last year. 30.6% report being emotionally exhausted from their work always and almost always. 15.3% report that working with patients every day is stressful for them.29.4% report feeling “burned” by work. Only 28.2% say that they are always or almost always with a lot of vitality. 20.2% feel that they are at the limit of their possibilities.

**Conclusions:**

Detection in hospitals of those people susceptible to exhaustion, work stress or personal demotivation to avoid a problem in the worker’s mental health, allowing early intervention and health strategies.

**Disclosure:**

No significant relationships.

